# Supplementary material for: VmsR, a LuxR-Type Regulator, Contributes to Virulence, Cell Motility, Extracellular Polysaccharide Production and Biofilm Formation in Xanthomonas oryzae pv. oryzicola
Source: Int J Mol Sci. 2024 Jul 11;25(14):7595. doi: 10.3390/ijms25147595 (PMC11277528; doi:10.3390/ijms25147595)
Supplement: Supplementary file 1 [file ijms-25-07595-s001.zip › ijms-3093529-supplementary/Figure S1.pdf]

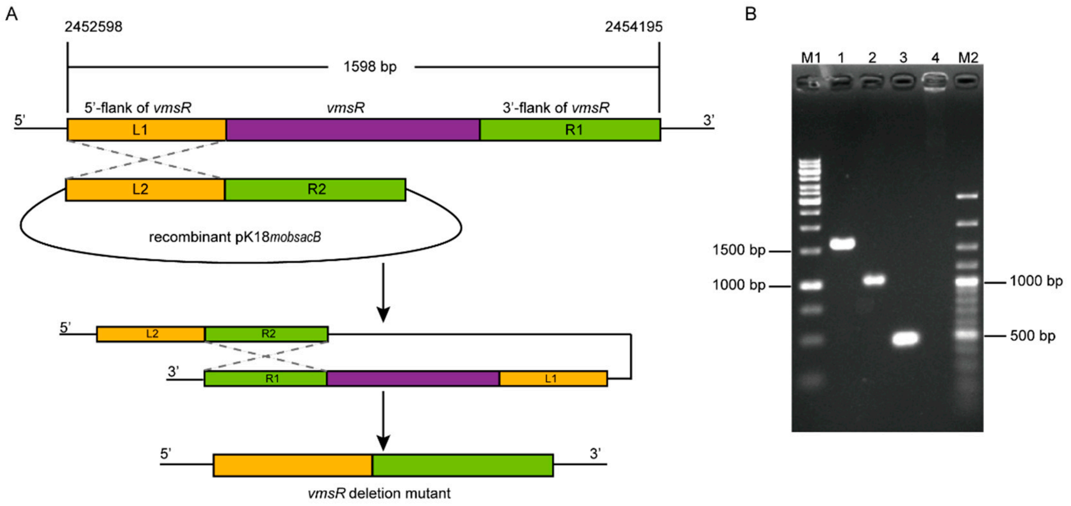

Figure S1: Construction of *vmsR* deletion mutant. (A) The model of homology double-swap. (B) The electrophoretic gel of *vmsR* mutant. M1: GeneRuler 1kb DNA Ladder. Lane 1: Validation of external primers using GX01 as a template. Lane 2: Validation of external primers using *vmsR* mutant as a template. Lane 3: Validation of internal primers using GX01 as a template. Lane 4: Validation of internal primers using *vmsR* mutant as a template. M2: GeneRuler 100b Plus DNA Ladder.
