# Supplementary material for: VmsR, a LuxR-Type Regulator, Contributes to Virulence, Cell Motility, Extracellular Polysaccharide Production and Biofilm Formation in Xanthomonas oryzae pv. oryzicola
Source: Int J Mol Sci. 2024 Jul 11;25(14):7595. doi: 10.3390/ijms25147595 (PMC11277528; doi:10.3390/ijms25147595)
Supplement: Supplementary file 1 [file ijms-25-07595-s001.zip › ijms-3093529-supplementary/Figure S2.pdf]

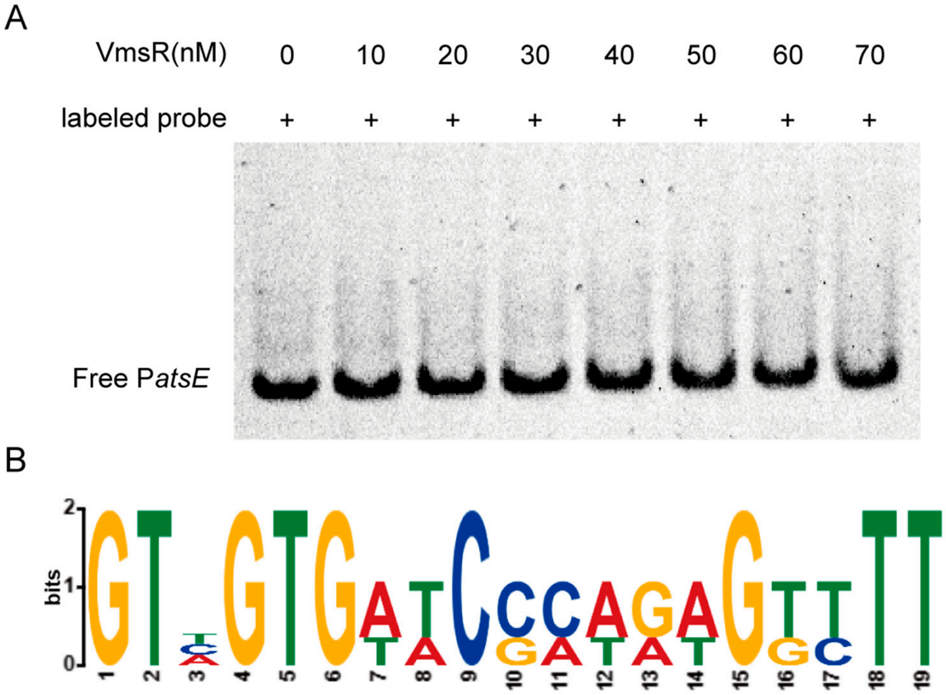

Figure S2: The EMSA between VmsR-His_6 and the negative control promoter (*atsE* promoter), and the putative binding motif. (A) The EMSA between VmsR-His_6 and *atsE* promoter. The reaction contained VmsR (10-70 nM) and 6′-FAM-labelled probes (~10 ng). (B) MEME-predicted conserved DNA sequence bound by VmsR.
