# Supplementary material for: VmsR, a LuxR-Type Regulator, Contributes to Virulence, Cell Motility, Extracellular Polysaccharide Production and Biofilm Formation in Xanthomonas oryzae pv. oryzicola
Source: Int J Mol Sci. 2024 Jul 11;25(14):7595. doi: 10.3390/ijms25147595 (PMC11277528; doi:10.3390/ijms25147595)
Supplement: Supplementary file 1 [file ijms-25-07595-s001.zip › ijms-3093529-supplementary/Table S1.pdf]

Table S1 Bacterial strains and plasmids used in this study.

Bacterial strain	Relevant characteristics	Reference or source
Xanthomonas oryzae* pv. *oryzicola		
GX01	Wild-type strain, Rif^r	Our laboratory
Δ*Xoc_2507*	*Xoc_2507* deletion mutant, Rif^r	This work
Δ*Xoc_2507*/*Xoc_2507*	*Xoc_2507* complementation strain, containing pXUK-*Xoc_2507*; Rif^r, Km^r	This work
Escherichia coli		
DH5α	F^-φ80d *lac*ZΔM15 Δ(*lac*ZYA-*arg*F)U169 *end*A1 *rec*A1 *hsd*R17(r_K^-, m_K^+) *sup*E44λ-*thi*-1 *gyr*A96 *rel*A1 *pho*A	TransGen Biotech
BL21(DE3)	F^- *omp*T *hsd*S_B(r_B- m_B-) *gal* *dcm*(DE3)	TransGen Biotech

Plasmids	Relevant characteristics	Reference or source
pK18*mobsacB*	Suicide plasmid in *X. oryzae* pv. *oryzicola*, Km^r	Our laboratory
pK-*Xoc_2507*-	pK18*mobsacB* carrying 493 bp	This work

up&down	upstream and 522 bp downstream sequences of *Xoc_2507*, Km^r	
pXUK	Derived from indigenous plasmid pXOCgx01 of *X. oryzae* pv. *oryzicola* GX01, Km^r	Our laboratory
pXUK-*Xoc_2507*	pXUK carries 633bp coding region of *Xoc_2507*, Km^r	This work
pRSFDuet-1	pRSFDuet-1 carries the RSF1030 replicon, *lacI* gene and kanamycin resistance gene.	EMD Biosciences (Novagen)
pRSFDuet-1-*Xoc_2507*	pRSFDuet-1 carries 633bp coding region of *Xoc_2507*, Km^r	This work

Note: Rif^r, rifampicin-resistant; Km^r, kanamycin-resistant.
