# Supplementary material for: VmsR, a LuxR-Type Regulator, Contributes to Virulence, Cell Motility, Extracellular Polysaccharide Production and Biofilm Formation in Xanthomonas oryzae pv. oryzicola
Source: Int J Mol Sci. 2024 Jul 11;25(14):7595. doi: 10.3390/ijms25147595 (PMC11277528; doi:10.3390/ijms25147595)
Supplement: Supplementary file 1 [file ijms-25-07595-s001.zip › ijms-3093529-supplementary/Table S2.pdf]

Table S2 Primers used in this study.

1. The primers of deletion mutant

Primers	Sequence	Restriction enzyme	Product of DNA
Xoc_2507-LF	CGGGATCCCGTAGCGAGGAAACCAGCGAA	*Bam*H Ⅰ	493 bp
Xoc_2507-LR	GCTCTAGAGTCGACGATGATGACTCGCA	*Xba* Ⅰ	
Xoc_2507-RF	GCTCTAGACGGCCAAACATCACAACGAA	*Xba* Ⅰ	522 bp
Xoc_2507-RR	CCCAAGCTTCATCGTTCCAACTGCTGCTC	*Hind*Ⅲ	
Xoc_2507-cmF	CCCAAGCTTGGTGCGAGTCATCATCGTCGACG	*Hind*Ⅲ	633 bp
Xoc_2507-cmR	GCTCTAGATCAGACCAATTCGTTGTGATGT	*Xba* Ⅰ	
Xoc_2507-inF	CCAGGCTGCTGCAAACTTTC	/	482 bp
Xoc_2507-inR	CTTGACGCTGATGCCCAGAT	/	
Xoc_2507-exF	CGGGATCCGTGCGAGTCATCATCGTCGACG	*Bam*H Ⅰ	633 bp
Xoc_2507-exR	CCCAAGCTTTCAGACCAATTCGTTGTGATGT	*Hind*Ⅲ	

Note: The underlined text denotes the enzyme cleavage sites, along with the guard bases.

2. The primers of RT-qPCR

Primers	Sequence	Product of DNA

Xoc_3054(*gumB*)-qF	TTGCAAAATCCGCAGGTGTC	185 bp
Xoc_3054(*gumB*)-qR	CGGAACACGATGACATTGCC	
Xoc_2502(*fliC*)-qF	AGAGCATCACCATCGCTGAC	125 bp
Xoc_2502(*fliC*)-qR	GCTCTTGATCGAGGCGTACA	
Xoc_2503(*fliD*)-qF	CAACAGCGTCACCGTGGATA	149 bp
Xoc_2503(*fliD*)-qR	CGGAATCGACCGCATTGAAG	
Xoc_2504(*fliS*)-qF	GCAATCGTCGGTCACTTGAA	124 bp
Xoc_2504(*fliS*)-qR	CATCGTTGTGCAAGTTGGCT	
Xoc_2617(*cheB*)-qF	TTCGTGTCCAAGCCCAAGAT	143 bp
Xoc_2617(*cheB*)-qR	TGCATATCGAGCGTGACCTT	
Xoc_2615(*cheR*)-qF	CTGACCACCAACCTGACCTC	149 bp
Xoc_2615(*cheR*)-qR	GTGATGGCGATGGAGTAGGG	
Xoc_2612(*cheW*)-qF	GCCCGACTACATCAAGGGTG	189 bp
Xoc_2612(*cheW*)-qR	TCGTCAGTGAGCGGAATCAC	
Xoc_2593(*cheA*)-qF	ATCTGCTCGAGCGCAATACC	179 bp
Xoc_2593(*cheA*)-qR	GATCAGCCCCTTGTCCAACT	
Xoc_2592(*cheY*)-qF	TCTTTCGCCCTTACCTCTGC	194 bp
Xoc_2592(*cheY*)-qR	GTGAGCATGAGCATCGGAGT	
Xoc_2590(*cheW*)-qF	CGACTCGGCCTTCGTTCTG	168 bp
Xoc_2590(*cheW*)-qR	CGTTCGGCGTGGATCTTGTC	

Xoc_2510(*flrA*)-qF	TCCGGTACAGGCAAAGAAGTC	129 bp
Xoc_2510(*flrA*)-qR	GTGGCCGAACAATTCGCTTT	
Xoc_2614(*mcp*)-qF	GGATTGCCGACGAAAACCTG	128 bp
Xoc_2614(*mcp*)-qR	GTGCTGCGCCACGATCTTCT	
Xoc_2610(*tsr*)-qF	CGGCATTGTTGGACACATCC	166 bp
Xoc_2610(*tsr*)-qR	ATTCTGCTTGACCGTGGAGG	
Xoc_2606(*mcp*)-qF	CTCAGCAACGTCTCGCTACA	142 bp
Xoc_2606(*mcp*)-qR	GCGACAGGGTATACCAACCC	
Xoc_2604(*mcp*)-qF	TGTCTTCGGTCAATCGCGAAA	182 bp
Xoc_2604(*mcp*)-qR	AATGGACTGCAACAGGCCC	
Xoc_2861(*mcp*)-qF	TTGTCCGAAGGCGATCTCAC	102 bp
Xoc_2861(*mcp*)-qR	TTGCGACAACTTTTGCACCA	

3. The primers of probe

Primers	Sequence	Product of DNA
gumB-F	ACGGTCTGTCATGGGCTTTT	440 bp
gumB-R	6’FAM-CCTCGATTGGGTGGGACAG	
fliC-F	CGAAGAGTTATACCAGCGCT	283 bp
fliC-R	6’FAM-TTCAGCGACATTACGTTGGT	
fliD-F	CTTGCAGTCAGCTGCCGCCGC	272 bp
fliD-R	6’FAM-GTTGCATTCCTCGTCTGGGCG	

fliS-F	GCCTGACCATCGACAAGGAT	500 bp
fliS-R	6’FAM-GGATGACTCCTCAATGGGCT	
