# Supplementary material for: VmsR, a LuxR-Type Regulator, Contributes to Virulence, Cell Motility, Extracellular Polysaccharide Production and Biofilm Formation in Xanthomonas oryzae pv. oryzicola
Source: Int J Mol Sci. 2024 Jul 11;25(14):7595. doi: 10.3390/ijms25147595 (PMC11277528; doi:10.3390/ijms25147595)
Supplement: Supplementary file 1 [file ijms-25-07595-s001.zip › ijms-3093529-supplementary/Table S3.pdf]

Table S3 Categorization of differentially expressed genes (DEGs).

Gene ID	Gene Name	Description	Log_2FC	*p* value
**Amino acids biosynthesis (5)**				
Xoc_1026	*ilvB*	acetolactate synthase catalytic subunit	-1.0791	0.00058
Xoc_3433	*lysAC*	aspartate kinase	1.5747	0.000253
Xoc_3842	*tdh*	L-threonine 3-dehydrogenase	1.316	5.39E-05
Xoc_0517	*trpG*	anthranilate synthase component II	-1.1727	0.000404
Xoc_1841	*trpB*	tryptophan synthase subunit beta	1.5904	0.001368
**Biosynthesis of cofactors, prosthetic groups, carriers (2)**				
Xoc_0211	*bioB*	biotin synthase	1.2176	0.000152
Xoc_2042	*bchE*	Mg-protoporphyrin IX monomethyl ester oxidative cyclase	-1.2018	0.000101
**Cell envelope and cell structure (10)**				
Xoc_0014	/	membrane protein	1.3547	0.000699
Xoc_0065	/	membrane protein	-1.0386	0.000839
Xoc_0464	/	membrane protein	1.1463	0.000587
Xoc_0804	*ompW*	membrane protein	-1.4298	3.94E-06
Xoc_3307	/	membrane protein	1.1062	0.000697
Xoc_3309	/	membrane protein	1.2145	0.000249
Xoc_3437	/	membrane protein	-1.1924	0.000115

Xoc_3636	/	membrane protein	1.4989	5.24E-05
Xoc_3782	*slp*	membrane protein	-0.98739	0.00132
Xoc_2810	/	N-acetylmuramoyl-L-alanine amidase	1.2015	0.000173
		**Cellular processes (18)**		
Xoc_2589	*parA*	chromosome partitioning protein ParA	-1.2689	5.29E-05
Xoc_2212	*mcp*	methyl-accepting chemotaxis protein	-1.0437	0.000825
Xoc_2478	*mcp*	methyl-accepting chemotaxis protein	-1.3856	1.14E-05
Xoc_2502	*fliC*	fliC flagellin	-1.2415	6.277E-05
Xoc_2503	*fliD*	fliD flagellar protein	-1.0216	9.184E-04
Xoc_2504	*fliS*	fliS flagellar protein	-1.2216	1.054E-04
Xoc_2590	*cheW*	chemotaxis protein	-1.4671	3.586E-06
Xoc_2602	*tsr*	tsr methyl-accepting chemotaxis protein	-1.082	7.665E-04
Xoc_2603	*tsr*	tsr methyl-accepting chemotaxis protein	-1.0832	8.584E-04
Xoc_2604	*mcp*	mcp chemotaxis protein	-1.8818	3.054E-09
Xoc_2605	*tsr*	tsr methyl-accepting chemotaxis protein	-1.0823	8.88E-04
Xoc_2606	*mcp*	mcp methyl-accepting chemotaxis protein	-1.6289	4.091E-07
Xoc_2610	*tsr*	tsr chemotaxis protein	-1.1686	3.418E-04

Xoc_2612	*cheW*	cheW purine-binding chemotaxis protein	-1.9541	1.27E-09
Xoc_2614	*mcp*	mcp methyl-accepting chemotaxis protein	-1.3457	2.426E-05
Xoc_2615	*cheR*	chemotaxis protein methyltransferase	-1.6386	4.917E-07
Xoc_2617	*cheB*	two-component system response regulatory protein	-1.1851	3.185E-04
Xoc_2861	*mcp*	mcp methyl-accepting chemotaxis protein	-1.0137	1.343E-03
		**Central intermediary metabolism (6)**		
Xoc_3115	*cynT*	carbonic anhydrase	-0.98593	0.001567
Xoc_0482	*speE*	spermidine synthase	1.8186	0.000135
Xoc_1233	*glgX*	glycogen debranching enzyme GlgX	1.2596	0.000626
Xoc_1819	/	Carboxymethylenebutenolidase	1.2196	0.000196
Xoc_2751	*dgoA*	2-dehydro-3-deoxy-6-phosphogalactonate aldolase	1.3474	0.000761
Xoc_0102	*ivd*	isovaleryl-CoA dehydrogenase	-1.0185	0.00103
		**Energy and carbon metabolism (6)**		
Xoc_0129	/	2-alkenal reductase	1.0246	0.001442
Xoc_2024	/	cytochrome C	-1.0517	0.000715
Xoc_2025	/	cytochrome C	-1.0798	0.000572
Xoc_2026	*cybB*	cytochromelike B561	-1.0697	0.001193

Xoc_2255	/	cytochrome C biogenesis protein CcsA	1.3463	0.000462
Xoc_4332	*aceE*	pyruvate dehydrogenase E1 component	1.2928	0.001117
**Fatty acid and phospholipid metabolism (6)**				
Xoc_0574	*accC*	acetyl-CoA carboxylase	1.0833	0.00073
Xoc_0576	*accB*	acetyl-CoA carboxylase	1.4413	5.96E-05
Xoc_0850	*fabB*	3-oxoacyl-ACP synthase	1.1655	0.000281
Xoc_2513	*fabH*	3-oxoacyl-ACP synthase	-1.38	2.24E-05
Xoc_3770	*fabG*	3-ketoacyl-ACP reductase	1.6371	5.06E-06
Xoc_3773	*fabH*	3-oxoacyl-ACP synthase	1.9995	3.72E-05
**Nucleotides metabolism (4)**				
Xoc_0509	*purC*	phosphoribosylaminoimidazole-succinocarboxamide synthase	1.3337	3.84E-05
Xoc_2181	*guaB*	inosine-5-monophosphate dehydrogenase	1.8009	0.000254
Xoc_2182	*guaA*	GMP synthase	1.2181	0.000549
Xoc_2644	*carA*	carbamoyl-phosphate synthase, small subunit	-1.113	0.000382
**Regulatory functions (5)**				
Xoc_0726	/	two-component system response regulator protein	1.6706	0.000108

Xoc_1035	/	AraC family transcriptional regulator	-2.4146	2.93E-13
Xoc_1603	/	regulatory protein	-1.7472	3.43E-08
Xoc_2510	*flrA*	sigma-54-dependent Fis family transcriptional regulator	-1.0851	0.000445
Xoc_2591	/	anti-sigma factor antagonist	-1.2541	8.12E-05
		**Replication and DNA metabolism (5)**		
Xoc_1802	*dnaB*	replicative DNA helicase	1.1201	0.000513
Xoc_2056	*parE*	DNA topoisomerase IV subunit B	1.3973	0.000109
Xoc_3066	*gyrA*	DNA gyrase subunit A	1.3993	0.000739
Xoc_3405	/	pirin	1.7343	0.000109
Xoc_1898	*uvrB*	excinuclease ABC subunit B	1.3582	0.000174
		**Transport (4)**		
Xoc_2511	/	aminotransferase	-1.0707	0.001313
Xoc_3469	/	competence protein ComEA	-1.9314	0.000582
Xoc_1036	/	TonB-dependent receptor	-1.7621	2.14E-08
Xoc_4138	/	TonB-dependent receptor	1.0129	0.001553
		**Translation (12)**		
Xoc_2165	/	50S ribosomal protein L36	1.0737	0.00152
Xoc_3774	*rpmF*	50S ribosomal protein L32	2.6594	0.000138

Xoc_3775	/	characterized ACR protein	1.7929	0.000228
Xoc_0491	*argS*	arginine--tRNA ligase	1.9561	1.03E-05
Xoc_2102	*cysS*	cysteinyl-tRNA synthetase	1.0469	0.001131
Xoc_3268	*leuS*	leucine-tRNA ligase	1.0415	0.001115
Xoc_1654	*slyD*	peptidylprolyl isomerase	-1.3957	1.43E-05
Xoc_3804	*ppiD*	peptidylprolyl isomerase	1.6534	3.53E-07
Xoc_2982	*htpG*	chaperone protein htpG	1.7751	0.000269
Xoc_1605	*degP*	periplasmic S1	-1.7018	6.85E-08
Xoc_3214	*pepO*	peptidase M13	1.4791	0.000147
Xoc_4288	/	peptidase M16	1.283	5.97E-05
		**Transcription (1)**		
Xoc_2508	*rpoN*	RNA polymerase sigma-54 factor	-1.4277	6.10E-06
		**Signal transduction (7)**		
Xoc_0778	/	two-component system sensor-response regulator hybrid protein	1.2194	0.000127
Xoc_2507		Two-component system response regulator, LuxR family	-5.4358	1.97E-39
Xoc_2509	*vemR*	two-component system response regulatory protein	-1.1819	0.000206

Xoc_2592	*cheY*	two-component system response regulatory protein	-1.408	1.112E-05
Xoc_2593	*cheA*	two-component system sensor protein	-1.6242	2.743E-07
Xoc_2802	/	putative signal protein with PAS, GGDEF and EAL domains	1.4959	1.87E-05
Xoc_2915	/	diguanylate cyclase; two-component system response regulatory protein with GGDEF domain	-1.1212	0.000559
		**Mobile genetic elements (2)**		
Xoc_3211	/	DDE endonuclease	-2.1247	2.81E-11
Xoc_0123	/	transposase-like protein	1.5302	0.000248
		**Pathogenicity and adaptation (11)**		
Xoc_0268	*hrpF*	type III secretion translocon protein HrpF	1.3204	0.000843
Xoc_0284	*hrpB1*	type III secretion protein HrpB1	1.787	9.00E-06
Xoc_0675	*xopAK*	type III effector protein XopAK (AvrRxo1)	1.4734	0.000418
Xoc_0700	*tal5*	TAL effector protein Tal4; type III effector protein	3.418	0.000247
Xoc_1316	*vgrG*	type VI secretion protein Rhs	1.7448	0.000324

Xoc_1319	/	type VI secretion protein Rhs	1.2775	0.000105
Xoc_0889	*rfbB*	spore coat protein	1.1541	0.000452
Xoc_4804	*atsE*	attachment protein	2.3776	0.00159
Xoc_0051	*mdoG*	glucan biosynthesis protein D	1.208	0.000548
Xoc_2583	/	superoxide dismutase	-1.2736	5.24E-05
Xoc_0983	/	TonB-dependent receptor	1.1031	0.000496
		**Undefined category (13)**		
Xoc_0319	/	methylamine utilization protein	1.3003	0.001046
Xoc_0681	/	YceI like family	1.7449	0.001413
Xoc_1315	/	peptidase M61	1.9291	5.31E-06
Xoc_1885	*grxD*	glutaredoxin	1.3436	0.000332
Xoc_2256	/	cytochrome C	1.2608	0.000209
Xoc_2840	*citG*	triphosphoribosyl-dephospho-CoA synthase	-1.0982	0.00051
Xoc_2844	*ugl*	glucuronyl hydrolase	-1.5433	9.41E-07
Xoc_2845	*citE*	citrate lyase subunit beta	-2.0588	1.08E-10
Xoc_2949	/	FAD-dependent oxidoreductase	1.6439	0.000445
Xoc_3247	*pmbA*	peptidase PmbA	-1.1605	0.000227
Xoc_3517	/	endoproteinase ArgC	1.6224	0.000639
Xoc_4080	/	protease	1.4149	1.07E-05

Xoc_4661	/	thioesterase-like protein	1.2483	0.000212
		**Hypothetical proteins (23)**		
Xoc_0075	/	conserved hypothetical protein	1.103	0.000593
Xoc_0122	/	conserved hypothetical protein	1.8112	0.000629
Xoc_0127	/	conserved hypothetical protein	1.8996	0.000695
Xoc_0538	/	conserved hypothetical protein	-1.17	0.001169
Xoc_0777	/	conserved hypothetical protein	1.5889	0.000714
Xoc_0813	/	conserved hypothetical protein	2.1832	0.001229
Xoc_1268	/	NAD synthetase	-1.0151	0.001111
Xoc_1444	/	conserved hypothetical protein	-1.7694	2.16E-06
Xoc_1683	/	conserved hypothetical protein	1.1017	0.001576
Xoc_1774	/	conserved hypothetical protein	-1.4694	2.95E-06
Xoc_2041	/	conserved hypothetical protein	-1.2133	8.99E-05
Xoc_2613	/	conserved hypothetical protein	-1.9537	1.77E-09
Xoc_2756	/	conserved hypothetical protein	-1.0009	0.001161
Xoc_2858	/	conserved hypothetical protein	1.7008	0.00057
Xoc_2950	/	conserved hypothetical protein	1.4697	0.001022
Xoc_3002	/	conserved hypothetical protein	-1.3466	6.32E-05
Xoc_3033	/	conserved hypothetical protein	1.2278	0.000594
Xoc_3308	/	conserved hypothetical protein	1.4729	6.03E-06

Xoc_3374	/	conserved hypothetical protein	-1.2396	6.12E-05
Xoc_3938	/	conserved hypothetical protein	-1.1351	0.000305
Xoc_4141	/	conserved hypothetical protein	-1.0365	0.001289
Xoc_4143	/	conserved hypothetical protein	1.5181	6.05E-06
Xoc_4396	/	conserved hypothetical protein	1.7657	4.05E-05
