# Supplementary material for: VmsR, a LuxR-Type Regulator, Contributes to Virulence, Cell Motility, Extracellular Polysaccharide Production and Biofilm Formation in Xanthomonas oryzae pv. oryzicola
Source: Int J Mol Sci. 2024 Jul 11;25(14):7595. doi: 10.3390/ijms25147595 (PMC11277528; doi:10.3390/ijms25147595)
Supplement: Supplementary file 1 [file ijms-25-07595-s001.zip › ijms-3093529-supplementary/Table S4.pdf]

Table S4: Nucleotide sequences and amino acid sequences.

> *vmsR* nucleotide sequence:

GTGCGAGTCATCATCGTCGACGATCACACCCTTGTCCGTGCCGGCCTGTCCAGGCTGC
TGCAAACTTTCGCCGGCATCGATGTCGTGGGCGAGGCGAGCAACGCGCAACAAGCCC
TGGACATGACGTCCCTGCACCGGCCCGACCTGGTGTTGATGGACCTGTCCCTGCCTGG
CCGTAGCGGCCTGGATGCCATGACCGACGTACTGCGCGCTGCGCCGCGCACGCATGT
GGTGATGATGTCGATGCACGACGACCCGGTGCACGTGCGCGATGCGCTCGATCGCGG
CGCTGTCGGCTTCGTGGTCAAGGACGCCGCACCGCTGGAACTGGAACTGGCGTTGCG
CGCTGCCGCTGCTGGTCAAGTGTTCCTGAGCCCGCAGATCTCGTCCAAGATGATTGCG
CCGATGCTCGGTCGCGAAAAGCCGGTCGGTATTGCCGCACTGTCGCCACGCCAGCGC
GAGATTCTGCGCGAAATTGGCCGCGGCCAAAGCAATAAGGAAATCGCCGCCGATCT
GGGCATCAGCGTCAAGACGGTGGAAACCCACCGCGCACGCATGATGGAATCGCTCG
GTTGCCGTCGCGCCAACGATCTGGTGCTGCTGGCGGCCAAACATCACAACGAATTGG
TCTGA

>VmsR amino acid sequence:

MRVIIVDDHTLVRAGLSRLLQTFAGIDVVGEASNAQQALDMTSLHRPDLVLMDLSLPGRS
GLDAMTDVLRAAPRTHVVMMSMHDDPVHVRDALDRGAVGFVVKDAAPLELELALRAA
AAGQVFLSPQISSKMIAPMLGREKPVGIAALSPRQREILREIGRGQSNKEIAADLGISVKTVE
THRARMMESLGCRRANDLVLLAAKHHNELV

> *xocR* nucleotide sequence:

ATGTTTGAAATTCTAGCCAGCCTGGGCCGCGATCTGCAGGCGTCGCAAACGCTCAAT
GGCTGCCTGGATCGGGTGTTTCGCGATGTCTGTGCGCTCGGCTTCCAGTCGTTGGTCTA
CGACTACGCACCGGTGCCGCTGAGCATGGAGGGCGCGCTGATCACGCCAACGGTGTT
CATGCAGCGCAATGCGCCAGGCGATATGCAGCATGTCTGGTGCGAGCACGGGTACTA
CCAACATGACCCCGTTCAGCAGCGTGCAGCGCGACGTACCACCCCGTTCGTATGGTC
GTACCGCACCGACGGCGATTGCGCTGGGGTGGAATATGTGGGTGGACAGCACCGGC
AAGTCACGCGTTACTTATGCGATAGCGGCATGGGTACCGGTGTCACCGTGCCGCTGC
ATCTGCCCGGTGGTGCGTTCGCCACCTTTAGCGGTGCGATTGATGCCGTGGCTGCGGA
AGCGCTGCGTCTGGCCGAGTCGCAGTTATTGCCCTTCTTGCTGCTGGCACACGCTTTCC
AGGCGCGTGCGCAGGAATTGCTGGACCCGCAGGAACGCCGCTGCCACTACATCGCAT
TGACCCGTCGCGAGCGCGAATGCCTGCAGTATTCGGCCAAAGGCCTGACCTCCAAAC
GTATCGCCGCGGCGCTCAACCGCTCCACCGCCACGGTGAACCTGCATCTGAATTCGG
CTGCCCGCAAACTGGGGGCACGTAACCGCGTGGAAGCGGTGGTGCGTGGTATGCACT
ATCGGTTGCTGGAGCCATAA

>XocR amino acid sequence:

MFEILASLGRDLQASQTLNGCLDRVFRDVCALGFQSLVYDYAPVPLSMEGALITPTVFMQR
NAPGDMQHVWCEHGYYQHDPVQQRAARRTTPFVWSYRTDGDCAGVEYVGGQHRQVT
RYLCDSGMGTGVTVPLHLPGGAFATFSGAIDAVAAEALRLAESQLLPFLLLAHAFQARAQE
LLDPQERRCHYIALTRRERECLQYSAKGLTSKRIAAALNRSTATVNLHLNSAARKLGARNR
VEAVVRGMHYRLLEP
